# Multiparametric Phenotyping of Circulating Tumor Cells for Analysis of Therapeutic Targets, Oncogenic Signaling Pathways and DNA Repair Markers

**DOI:** 10.3390/cancers14112810

**Published:** 2022-06-06

**Authors:** Stephanie Staudte, Konrad Klinghammer, Philipp Sebastian Jurmeister, Paul Jank, Jens-Uwe Blohmer, Sandra Liebs, Peter Rhein, Anja E. Hauser, Ingeborg Tinhofer

**Affiliations:** 1 Department of Radiooncology and Radiotherapy, Charité University Hospital, 10117 Berlin, Germany; ingeborg.tinhofer@charite.de; 2 German Cancer Consortium (DKTK) Partner Site, and German Cancer Research Center (DKFZ), 69120 Heidelberg, Germany; 3 Department of Hematology and Oncology, Charité University Hospital, 10117 Berlin, Germany; konrad.klinghammer@charite.de; 4 Charité Comprehensive Cancer Center (CCCC), Charité University Hospital, 10117 Berlin, Germany; sandra.liebs@charite.de; 5 Institute of Pathology, Charité University Hospital, 10117 Berlin, Germany; philipp.jurmeister@med.uni-muenchen.de; 6 Institute of Pathology, Ludwig Maximilians University Hospital Munich, 80337 Munich, Germany; 7 Institute of Pathology, Philipps-University Marburg and University-Hospital Marburg (UKGM), 35039 Marburg, Germany; paul.jank@uni-marburg.de; 8 Breast Cancer Center, Charité University Hospital, 10117 Berlin, Germany; jens.blohmer@charite.de; 9 Luminex B.V., A DiaSorin Company, 5215 MV‘s-Hertogenbosch, The Netherlands; prhein@luminexcorp.com; 10 Department of Rheumatology and Clinical Immunology, Charité University Hospital, 10117 Berlin, Germany; hauser@drfz.de; 11 Deutsches Rheuma-Forschungszentrum (DRFZ), Leibniz Association, 10117 Berlin, Germany

**Keywords:** liquid biopsy, circulating tumor cells, imaging flow cytometry, Amnis^®^, multiparametric phenotyping, head and neck squamous cell carcinoma, breast cancer

## Abstract

**Simple Summary:**

Detection of circulating tumor cells (CTCs) has been established as an independent prognostic marker in solid cancer. In order to expand the clinical utility of this blood–based minimally invasive biomarker we established a protocol allowing multiparametric phenotyping of CTCs to analyze the expression levels of therapeutic target proteins. By applying this assay, we demonstrated intratumoral heterogeneity of PD–L1 expression in CTCs from head and neck cancer patients, an observation previously reported in tumor tissue specimens. We further verified the feasibility of applying the protocol to analyze the activation status of important oncogenic pathways and the extent of DNA repair following radiation. These promising preliminary results warrant further study and may lead to the implementation of this assay in clinical routine for improved treatment selection and monitoring.

**Abstract:**

Detection of circulating tumor cells (CTCs) has been established as an independent prognostic marker in solid cancer. Multiparametric phenotyping of CTCs could expand the area of application for this liquid biomarker. We evaluated the Amnis^®^ brand ImageStream^®^X MkII (ISX) (Luminex, Austin, TX, USA) imaging flow cytometer for its suitability for protein expression analysis and monitoring of treatment effects in CTCs. This was carried out using blood samples from patients with head and neck squamous cell carcinoma (*n* = 16) and breast cancer (*n* = 8). A protocol for negative enrichment and staining of CTCs was established, allowing quantitative analysis of the therapeutic targets PD–L1 and phosphorylated EGFR (phospho–EGFR), and the treatment response marker γH2AX as an indicator of radiation–induced DNA damage. Spiking experiments revealed a sensitivity of 73% and a specificity of 100% at a cut–off value of ≥3 CTCs, and thus confirmed the suitability of the ISX-based protocol to detect phospho–EGFR and γH2AX foci in CTCs. Analysis of PD–L1/–L2 in both spiked and patient blood samples further showed that assessment of heterogeneity in protein expression within the CTC population was possible. Further validation of the diagnostic potential of this ISX protocol for multiparametric CTC analysis in larger clinical cohorts is warranted.

## 1. Introduction

Circulating tumor cells (CTCs) represent the fraction of tumor cells which have detached from the tumor bulk and entered into blood circulation. Since they have the potential to travel to distant organs and seed new lesions, they are considered key players in metastasis formation [1]. The poor prognostic value of CTCs was first demonstrated in metastatic breast cancer [2] and was confirmed in subsequent studies in various solid cancers including colorectal [3], prostate [4], and head and neck cancer [5].

For the implementation of CTC detection as a blood–based biomarker in clinical routine, robust techniques for their enrichment and detection are prerequisites. The first system satisfying such requirements for use in clinical routine was the CellSearch^®^ (CS) platform (Huntington Valley, PA, USA). This FDA–approved automated CTC detection system captures CTCs via magnetic beads coupled to an antibody specific for epithelial cell adhesion molecule (EpCAM) [6]. Although representing a highly sensitive device for patients with EpCAM high expressing tumors, the CS system has its limitations in tumors consisting of cells which have undergone partial or complete epithelial to mesenchymal transition (EMT) [7], thereby displaying reduced or absent EpCAM expression. Another limitation is the equipment of the CS system, which has only four fluorescence channels, thus making it unsuitable for comprehensive phenotyping of CTCs. This represents a major constraint for further development of CTC assays, such as their use for the identification of therapeutic targets, the short–term evaluation of therapy response and early detection of disease progression.

In the present study, we evaluated the suitability of the Amnis^®^ ISX (Luminex, Austin, TX, USA), an imaging flow cytometer which can be equipped with up to ten fluorescence channels for CTC detection and multiparametric phenotyping. In addition to the assessment of sensitivity and specificity, we developed protocols for target identification (programmed cell death ligands 1 and 2 [PD–L1/–L2]; activated epidermal growth factor receptor; [EGFR]) and therapy response. For the latter, we focused on the assessment of the phosphorylated form of the histone 2a variant (γH2AX) as a surrogate marker of DNA repair efficacy after irradiation. As proof–of–concept, we used the ISX system for the analysis of blood samples from patients with head and neck squamous cell carcinoma (HNSCC) and breast cancer (BC).

## 2. Materials and Methods

### 2.1. Cell Lines

The cell lines used for spiking experiments were selected according to their expression levels of EpCAM and PD–L1/–L2. The cell lines SW620 (ATCC^®^ CCL 227™, purchased from ATCC, Manassas, VA; USA) and UD–SCC–4 (University of Düsseldorf, NRW, Germany) were used to determine the sensitivity of the CTC assay. The MDA–MB–231 cell line (ATCC^®^ HTB–26™, purchased from ATCC) was used to set the laser power and compensation matrix. For the establishment of the analysis of phospho–EGFR and γH2AX foci, UM– (University of Michigan, IL, USA) –SCC–22B, a gift from T.K. Hoffmann (University of Ulm, BW, Germany) [8] and the FaDu cell line (ATCC^®^HTB–43™, purchased from ATCC) were used, respectively. Cell cultures were maintained in a humidified incubator at 37 °C and 5% CO_2_. The composition of cell culture media is described in Supplementary Table S1.

### 2.2. Blood Collection

For our pilot study, healthy donors (*n* = 7), as well as HNSCC (*n* = 16) and BC patients (*n* = 8) presenting at the Charité for tumor treatment were included. After obtaining informed consent, blood samples were collected in ethylenediaminetetraacetic acid (EDTA) coated vacutainer tubes (BD, NJ, USA) and stored at room temperature (RT) for at least 30 min before further processing.

### 2.3. Blood Sample Processing for CTC Enrichment

Blood samples were processed within four hours after blood withdrawal. Leukocyte depletion was performed using the RosetteSep™ Human CD45 Depletion Cocktail (Stemcell Technologies, Vancouver, BC, Canada) according to the manufacturer’s instructions for 50 mL standard tubes. Briefly, after incubation with 50 µL of Depletion Cocktail per milliliter blood for 20 min at RT, blood was diluted with an equal volume of buffer composed of Dulbecco’s Phosphate Buffered Saline (PBS; Gibco™, Waltham, MA, USA, cat. no. 14190–094) and 2% Fetal Bovine Serum (FBS; Gibco™, cat. no. 10270–106). Ficoll–Paque™ PLUS (Cytiva, Marlborough, MA, USA) was overlaid with diluted blood and centrifuged at 1200× *g* for 20 min at RT with the break off. The interphase, consisting of peripheral blood mononuclear cells (PBMCs) and CTCs/spiked tumor cells between plasma and ficoll, was harvested using a Pasteur pipette and directly transferred into a new 50 mL tube, which was then filled up with buffer (PBS/2%FBS). The washing procedure was performed twice and cells were centrifuged at 300× *g* for 10 min at RT with low break. Finally, the cell pellet was suspended in staining buffer (PBS/10%FCS) and transferred into a 5 mL FACS tube for further staining (Supplementary Figure S1).

### 2.4. Immune Fluorescence (IF) Staining

The following fluorescence–labelled antibodies were used for CTC phenotyping: AlexaFluor^®^ 488 anti–human CD326 (EpCAM) Antibody (Biolegend, San Diego, CA, USA, cat.no. 324210, clone: 9C4, 1:100); AlexaFluor^®^ 488 anti–human EGFR Antibody (Biolegend, cat. no. 352908, clone: AY13, 1:100); AlexaFluor^®^ 647 anti–human CD45 Antibody (Biolegend, cat. no. 304018, clone: HI30, 1:50); APC/Fire™ 750 anti–human CD45 Antibody (Biolegend, cat. no. 304062, clone: HI30, 1:50); PE anti–human CD274 (B7H1, PD–L1) Antibody (Biolegend, cat. no. 393608, clone: MIH2, 1:20); PE–Vio^®^ 770 anti–human CD273 (PD–L2) REAfinity™ Antibody (Miltenyi, Bergisch Gladbach, NRW, Germany, cat. no. 130–116–565, clone: REA985, 1:50).

After centrifugation of cell suspension at 300× *g* for 5 min at 4 °C, FcR Blocking reagent (Miltenyi, cat. no. 130–059–901, 1:10) was added and samples were incubated for 10 min. All further steps were performed protected from light. Cells were incubated with the directly fluorescence–conjugated antibodies for extracellular staining for 15 min at 4 °C. After washing with 2 mL of staining buffer, cells were fixed with 4% formaldehyde (Carl Roth, BW, Germany, cat. no. 4979.1, 1:9.25 dilution of 37% formaldehyde) for 15 min and counterstained with Hoechst 33342 (LifeTechnologies, Waltham, MA, USA, cat. no. H1399, 2 ng/mL) for 20 min.

For the establishment of the protocol for phospho–EGFR and γH2AX foci detection, cells were treated with 100 ng/mL EGF (Invitrogen, Waltham, MA, USA, cat. no. PHG0315) for 10 min at 37 °C and 5% CO_2_ and/or irradiated with 2 Gray (Gy), respectively. After irradiation, cells were cultivated for 1 h and then harvested by trypsin treatment. For combined staining of surface and intracellular markers, the modified protocol of Durdik et al. [9] was used. Briefly, intracellular staining cells were fixed with 3% formaldehyde for 10 min at 4 °C, washed twice with 1 mL PBS, resuspended in 70% Ethanol and stored at −20 °C overnight. After an additional washing step, cells were permeabilized for 30 min at 4 °C by adding PBS supplemented with 1% BSA and 0.1% Triton X–100 (Th. Geyer, Renningen, BW, Germany). The following primary and secondary antibodies were used: Phospho–EGF Receptor (Tyr1068) (D7A5) XP^®^ Rabbit mAb (Cell Signaling, Danvers, MA, USA, cat. no. 3777S, 1:1600); Texas Red–labelled goat anti–Rabbit IgG (Invitrogen, cat. no. T–2767, 4 µg/mL); AlexaFluor^®^ 647 anti H2A.X Phospho–Ser139 Antibody (Biolegend, cat. no. 613408, clone: 2F3, 1.25 µg/mL). Samples were incubated with the primary antibodies for 2 h at RT. For secondary staining, cells were washed by centrifugation at 300× *g* for 5 min, resuspended in PBS and incubated with the antibody for 1 h at RT. After one additional washing step, cells were resuspended in 30 µL PBS and transferred into a 1.5 mL tube for imaging flow cytometric analysis.

### 2.5. Imaging Flow Cytometry—Amnis^®^ ImageStream^®^X Mk II

The ISX equipped with lasers at 405 nm, 488 nm, 561 nm and 642 nm and the INSPIRE™ software (version 201.1.0.765; Luminex, Austin, TX, USA) was used for sample acquisition. Data analysis was performed with the IDEAS^®^ software (version 6.2.187; Luminex, Austin, TX, USA). Cells were imaged at 40× magnification at low speed for receiving high–quality images with an acquisition time of 30 min per patient sample. The optimal compensation matrix between individual fluorescence channels was established using a mixture of MDA–MB–231 cells and PBMCs from a healthy donor, stained with each of the above–mentioned antibodies separately or in combination. The settings for acquisition and analysis were used for all samples (Supplementary Table S2).

### 2.6. Spiking Experiments

SW620 and UD–SCC–4 cells were harvested at a confluence of 90% by trypsin treatment. After cell counting using a conventional Neubauer Counting Chamber, aliquots of the cell suspension containing 500, 50 or 5 cells were prepared and added to 4 mL of blood from a healthy donor (blood–spiked samples) or to 4 mL of culture medium (reference samples). The blood–spiked samples were further processed using the RosetteSep^TM^ Human CD45 Depletion Cocktail (Stemcell Technologies) as described above. Reference samples were used to calculate the recovery rate of the CTC assay. Staining and acquisition were performed as described above.

## 3. Results

### 3.1. Establishment of Multicolor CTC Detection

**Figure 1:**
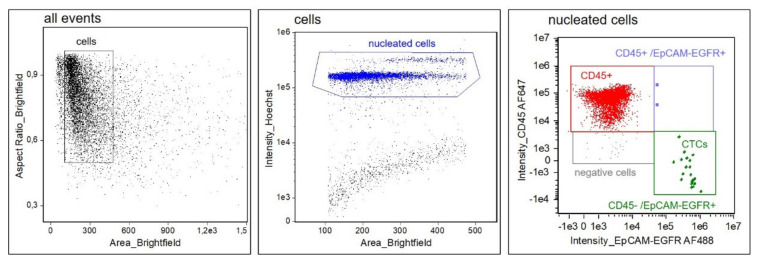
**Gating strategy for CTC detection.** At first, speedbeads and debris are excluded. Hoechst staining intensity of the gated events is displayed in a 2D plot. Hoechst^positive^ nucleated cells (blue) are then separated by their fluorescence intensities for EpCAM–EGFR–AF488 and CD45–AF647. CTCs are defined as Hoechst^positive^/CD45^negative^/EpCAM–EGFR^positive^. Single CTCs are detected in the CD45^negative^/EpCAM–EGFR^positive^ gate (green), whereas cluster of leukocytes and CTCs are detected in the CD45^positive^/EpCAM–EGFR^positive^ gate (purple).

Since EpCAM expression was shown to be downregulated in squamous cell carcinomas [10], whereas EGFR is frequently overexpressed in HNSCC [11], we hypothesized that the inclusion of both tumor–associated markers may increase sensitivity for the detection of CTCs, especially in tumors with an EMT phenotype displaying low or absent EpCAM expression. After identifying the optimal antibody concentrations, the following gating strategy was developed (Figure 1): after the exclusion of speedbeads and debris, a gate was set on Hoechst^positive^ nucleated cells and fluorescence intensities of CD45–AF647 and EpCAM–EGFR–AF488 were visualized in a 2D dot plot. CTCs were detected within the CD45^negative^/EpCAM–EGFR^positive^ gate or, when clustered to leukocytes, in the CD45^positive^/EpCAM–EGFR^positive^ gate. This CTC gating strategy was then applied to all subsequent experiments.

**Figure 2:**
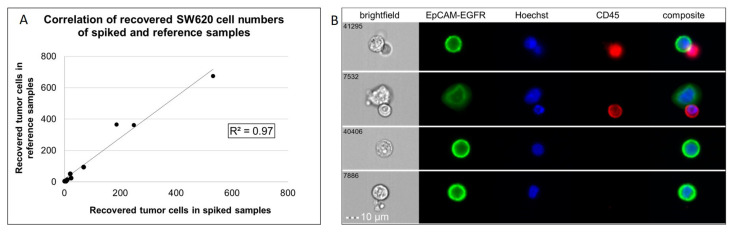
**Detection of spiked SW620 cells by imaging flow cytometry.** (**A**) Analysis of samples (*n* = 3 replicates per dilution) revealed a correlation of R^2^ = 0.97 of recovered cells in reference and spiked samples. (**B**) Representative images from a spiking experiment.

Spiking experiments were performed to investigate the sensitivity and specificity of the ISX–based assay. The procedure of the spiking experiments, including staining for CTC detection and ISX acquisition, is described in the Material and Methods section. Recovery frequencies (%–RF) were calculated by dividing the number of tumor cells detected in the blood–spiked sample by the number of tumor cells detected in the reference sample, multiplied by 100. The median recovery of three independent technical replicates of spiked SW620 cells was 73%. Overall, a correlation of R^2^ = 0.96 (SW620) was observed for recoveries from spiking samples (Figure 2A). Similar results were received for UD–SCC–4 (R^2^ = 0.92; Supplementary Figure S2). We also confirmed that our protocol allowed for a clear discrimination between single CTCs (Hoechst^positive^/EpCAM–EGFR^positive^/CD45^negative^) and CTC–leukocyte clusters (Hoechst^positive^/EpCAM–EGFR^positive^/CD45^positive^) (Figure 2B).

Blinded analysis of blood samples (*n* = 11) from healthy donors (*n* = 7) was performed in order to determine the specificity of the CTC detection assay. For a positive control, 500 UD–SCC–4 cells were spiked into a blood sample immediately before sample blinding. Staining and ISX analysis were performed as described above. Subsequently, in 1 of the 11 healthy donor samples (9%), two EpCAM–EGFR^positive^ cells were detected. Thus, without applying a threshold, a specificity of 91% was achieved by our protocol. A specificity of 100% was obtained when applying the cut–off value of ≥3 CTCs, a threshold previously associated with a significantly worse outcome in metastatic colon cancer [3].

### 3.2. Phenotyping of CTCs

The ISX–based protocol was further developed for multiparametric phenotyping of CTCs. Considering their important role as therapeutic targets in various epithelial tumor entities, EGFR in its phosphorylated activated form (phospho–EGFR) and the two immune checkpoints PD–L1 and PD–L2 were included in the marker panel. In addition, for analysis of residual DNA double strand breaks as a potential marker of radioresistant CTCs emigrating from the irradiated field, we also included detection of the phosphorylated form of the histone 2a variant (γH2AX) to our multiparametric panel.

In the first step, the staining was set up for each single marker. Phospho–EGFR staining was established using the UM–SCC–22B cell line displaying high EGFR expression levels. Cells were left untreated (negative control) or were treated with EGF (positive control). PD–L1/–L2 staining was established with the cell lines MDA–MB–231 (PD–L1^positive^ PD–L2^weak^) and SCC–25 (PD–L1^weak^ PD–L2^positive^). For establishment of γH2AX foci staining, FaDu cells were left untreated (negative control), or were irradiated with a single dose of 2 Gy or 10 Gy (positive controls). Isotype controls were included for assessment of background signals, except for phospho–EGFR staining in which staining with the secondary antibody alone was used to determine unspecific signals.

**Figure 3:**
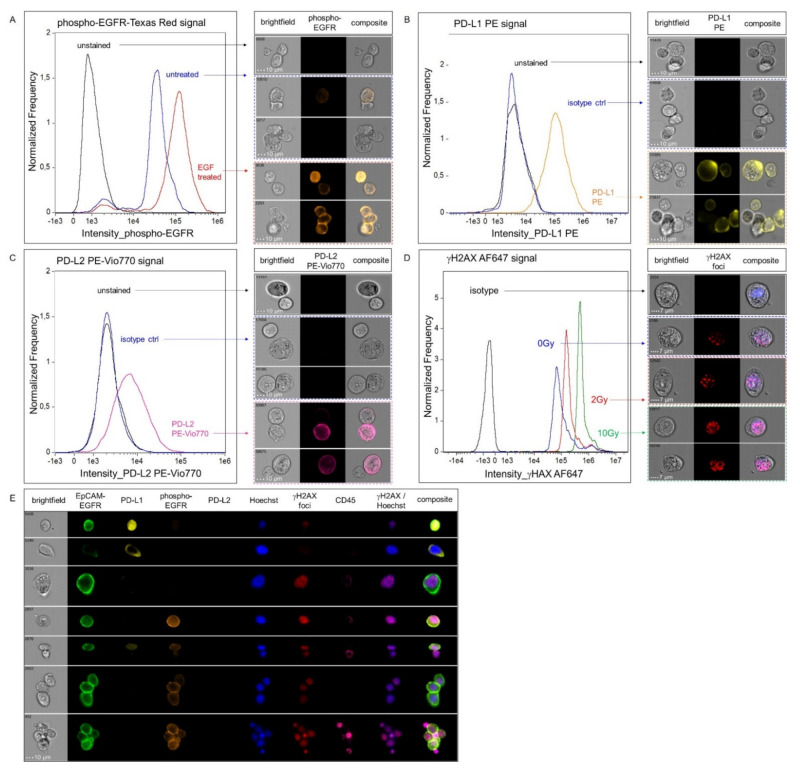
**Immunofluorescence analysis of phospho**–**EGFR, PD**–**L1/PD**–**L2 and γH2AX and multiparametric phenotyping**. (**A**) Representative results of phospho–EGFR staining in UM–SCC–22B cells are shown. The histogram displays the fluorescence intensities in unstained (black), untreated (blue) and EGF–treated (red) cells. (**B**,**C**) Positive and negative cells for surface staining of PD–L1 (B) and PD–L2 (C) on MDA–MB–231 and SCC–25 cells, respectively, are shown. Fluorescence intensities for unstained (black), isotype control (blue) or samples stained with either anti–PD–L1 (orange) or anti–PD–L2 antibody (pink) are depicted. Clusters of cells were selected to demonstrate the heterogeneity of each cell line and the feasibility of the ISX to detect different expression intensities with high sensitivity. (**D**) γH2AX staining was performed in FaDu cells, untreated (blue) or irradiated with 2 Gy (red) or 10 Gy (green). Isotype control is shown in black. Representative images for the different conditions (acquired with the 60× objective at low speed) are presented at the right column of the figure. (**E**) Multiparametric phenotyping of spiked EGF–treated and irradiated FaDu cells in peripheral blood (acquired with 40× magnification).

Analysis of each marker individually confirmed that a clear discrimination of untreated and EGF–treated (Figure 3A) and PD–L1/–L2 negative versus positive cells was possible (Figure 3B,C). In line with the successful detection of DNA double strand breaks, a dose–dependent increase in the number of γH2AX foci (Supplementary Figure S3) and in the nuclear γH2AX signal intensity was observed in irradiated compared to non–irradiated cells (Figure 3D). The whole antibody panel was validated by spiking FaDu cells, which had previously been irradiated and treated with EGF, into blood samples from a healthy donor. The suitability of our protocol for parallel analysis of EGFR signaling activity, immune checkpoint expression and DNA damage response was thereby confirmed (Figure 3E).

### 3.3. Assessment of CTCs in Blood Samples from Patients with HNSCC and BC

In order to test the suitability of our ISX protocol for multiparametric CTC phenotyping in patients with epithelial cancer, blood samples from patients with HNSCC (*n* = 16) and BC (*n* = 8) were used. Patient characteristics are presented in Table 1.

**Figure 4:**
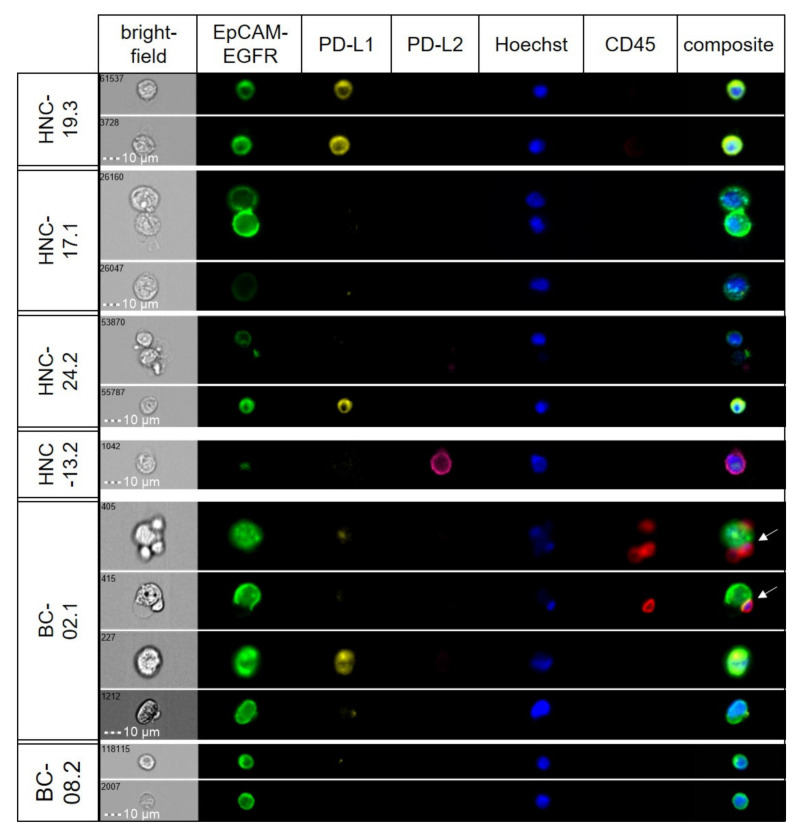
**Detection of intratumoral heterogeneity in marker expression in CTCs**. Representative images from selected patient samples (head and neck; HNC), showing varying expression levels of target proteins. In one case (BC–02.1), clusters of CTCs and leukocytes were observed (white arrows).

Applying the protocol for surface marker analysis and using a cut–off value for CTC–positivity of ≥3 CTCs, 7 out of 16 HNSCC patients tested positive for CTCs. In 4 of the CTC^positive^ cases PD–L1 expressing CTCs were detected and the amount of PD–L1^positive^ CTCs ranged from 56% to 100%. In the BC cohort, ≥3 CTCs were found in 6 out of 8 patients. Here, PD–L1 expression was detectable in 67% of the CTC^positive^ cases. Its expression varied from being absent to high within individual samples (Figure 4).

**Figure 5:**
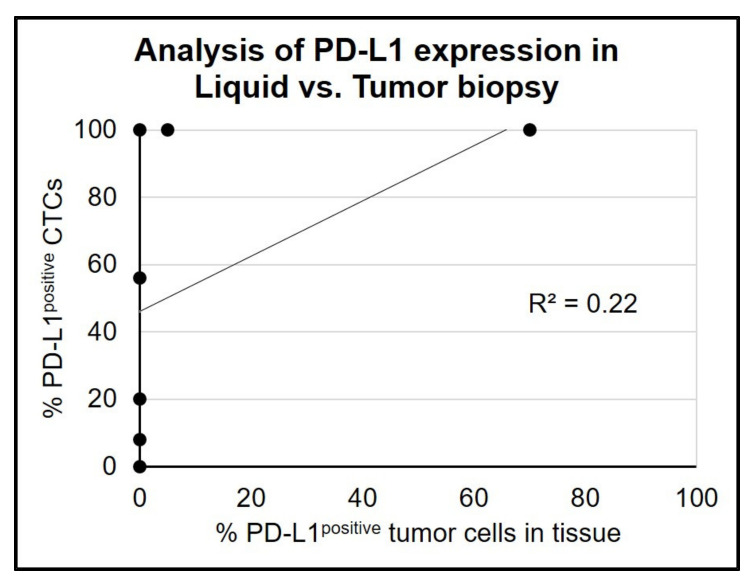
**Paired analysis of PD**–**L1 expression in tumor tissue and liquid biopsy.** Cases with ≥3 CTCs detected at baseline were used for paired analysis of PD–L1 expression analysis in tumor tissue and corresponding liquid biopsy. (pairs *n* = 7; BC: *n* = 2, HNSCC: *n* = 5).

In the light of the substantial inter– and intrapatient tumoral heterogeneity in PD–L1 expression in CTCs, we next determined the correlation between liquid and tumor biopsies. Paired analysis of PD–L1 expression on CTCs and the corresponding tumor tissue in seven cases (HNSCC *n* = 5; BC *n* = 2) revealed a weak correlation (R^2^ = 0.22; Figure 5).

A concordance between tumor tissue and liquid biopsy was found in four cases, where in two patients with PD–L1 expressing tissues (tumor proportion score 5% and 70%) all corresponding CTCs were PD–L1^positive^. The other two concordant cases were negative for PD–L1 in both sample types. The remaining cases had detectable PD–L1 expression on CTCs, but the corresponding tumor tissue was tested negative for PD–L1. The median time between tissue collection and liquid biopsy was 4.6 months (range 0.4 –19 months, Table 2), where the conventional biopsy was carried out first.

## 4. Discussion

To date, only a few studies have assessed the suitability of the ISX system for CTC detection [12,13,14,15,16]. In the current study, we could extend this limited evidence and show that sensitive and specific CTC detection is feasible by using this platform. Spiking experiments with our ISX protocol showed a median recovery rate of 73%, which was similar to the ISX–based CTC study of Ruiz-Rodríguez et al. [12], and even slightly better compared to other ISX–based studies showing recovery rates from 44% to 55%, respectively [13,14]. We did not compare the performance of the ISX with the CS system on corresponding patient samples. Evidence from the literature, however, strongly supports equivalent performance of both platforms, which is also underlined by results from a head–to–head comparison in the study of López–Riquelme et al. [17]. Using blood samples spiked with the pancreatic cancer cell line PANC–1 and staining for the tumor–cell markers EpCAM and cytokeratin, similar detection rates for the CS and ISX systems were observed at spiked cell numbers >10 [17]. In contrast to this study, where lower sensitivity of the ISX was reported for ≤10 spiked cells, we observed robust detection of spiked tumor cells even at a minimum of 5 cells per 4 mL blood. Combined staining of EpCAM and EGFR as tumor–associated markers might explain the high sensitivity of our ISX protocol.

Without applying a cut–off for CTC positivity, the specificity of our ISX protocol was 91%, where 1 of 11 samples tested positive in a blinded analysis of healthy donor blood samples. While the optimal cut–off for definition of a poor–outcome group has yet to be established in HNSCC [5,18], a cut–off of ≥3 CTCs/7.5 mL blood has been demonstrated to identify colon cancer patients with poor outcomes [3]. Using this threshold, specificity increased to 100% for our CTC detection protocol. Again, this high specificity is in line with the results from previous ISX–based CTC studies [14,16].

We further demonstrated that the ISX platform can be exploited for quantitative analysis of EGFR pathway activity in CTCs. Inclusion of phospho–EGFR in multiparametric CTC analysis is promising given the interference of EGFR signaling with DNA repair [19] and radioresistance [20]. Furthermore, Serrano et al. have shown that EGFR^positive^ CTCs co-express EMT markers, indicative of a high metastatic potential of this CTC subpopulation [21]. Induction of EMT by radiotherapy has been suggested as the underlying mechanism of the increase in CTC numbers observed in patients treated with radiotherapy [22]. Assessment of γH2AX foci as a marker of radiation–induced DNA double strand breaks [23] using our ISX protocol might represent an interesting biomarker by allowing for a discrimination between CTCs derived from the bulk tumor treated by radiotherapy and those originating from micrometastases outside of the irradiated field. By spiking experiments with irradiated and non–irradiated cells, we showed that the semi–automated quantification of dose–dependent effects of radiation in CTCs is possible by using our protocol. Dynamic assessment of viable CTCs derived from the radiation field and persisting during radiotherapy may not only identify radioresistant tumors [24,25,26] but could also guide the clinical development of combined therapies with radiosensitizing drugs [27,28].

Immune checkpoint inhibitors (ICIs) are currently developed in the curative and recurrent/metastatic setting. Immunohistochemical staining of PD–L1 in tumor tissue has been established as a predictive marker of treatment efficacy of PD–1 inhibitors. However, intratumoral heterogeneity in PD–L1 expression [29,30] can limit the accuracy of this biomarker, and could explain why some patients with PD–L1^negative^ tumors respond to ICIs while others with PD–L1 expressing tumors do not benefit from this treatment [31,32]. Thus, complementary analysis of PD–L1 expression in tumor and liquid biopsies could improve the predictive value of PD–L1. Preliminary evidence from the analysis of PD–L1 in CTCs in melanoma [32] and non–small cell lung cancer (NSCLC) [33,34] supports this hypothesis. In melanoma, detection of PD–L1^positive^ CTCs at baseline was associated with a significantly longer progression–free survival after pembrolizumab treatment [32]. A similar observation was reported for patients with NSCLC treated with nivolumab [34]. In both studies, intrapatient heterogeneity of PD–L1 expression in CTCs, and a low to moderate correlation between PD–L1 status of CTCs and tumor tissue, were reported, in line with the preliminary results of our study. In contrast, while basal PD–L1^positive^ CTC numbers were not associated with nivolumab efficacy in a study of advanced NSCLC, their persistence after treatment identified patients with decreased progression-free survival [35,36]. This negative prognostic value of the presence of PD–L1 expressing CTCs after treatment was also shown by Tan et al. in a mixed cohort of patients with advanced cancers [37]. In addition to the accumulating evidence of a positive correlation between baseline PD–L1^positive^ CTCs and the response to ICI treatment, a negative association with survival after treatment with other non–ICI regimes has been demonstrated in HNSCC [36] and NSCLC [38].

We confirmed the applicability of our assay in a small cohort of HNSCC (*n* = 16) and BC (*n* = 8) patients. The application of a cut–off value for CTC–positivity of ≥ 3 CTCs resulted in 7 out of 16 (44%) HNSCC patients being classified as CTC^positive^ at baseline. Similar detection rates by the CS system have been reported from previous HNSCC studies [5,39]. Among the seven CTC^positive^ cases, 57% had PD–L1 expressing CTCs. This was higher than reported by Strati et al. who could detect PD–L1 expression in 12% of the CTC^positive^ cases [36], however, the small size of the cohorts did not allow a statistical comparison of the two studies.

Concordance between PD–L1 expression in tumor tissue and CTCs was weak in our study. Discrepant results were reported for lung cancer, ranging from no/low [33,40] to high concordance [41]. A low correlation of PD–L1 expression in tumor tissue and liquid biopsy could be due to spatial heterogeneity [29,30,42] and/or dynamic changes in PD–L1 expression in tumors which cannot be captured by a single tissue biopsy. Also, the impact of time and/or treatment applied between tissue and liquid biopsy collection on the concordance of PD–L1 expression remains unclear. Future studies that include larger patient numbers will certainly be needed to establish the extent of intratumoral heterogeneity in PD–L1 expression and the complementary value of CTC–based PD–L1 analysis as a predictive marker of ICI efficacy.

Indeed, one major limitation of our study was the small number of patient samples. In order to establish the predictive and prognostic value of the CTC biomarker panel, analyses in larger cohorts of HNSCC patients with locally advanced disease treated with radiotherapy as well as recurrent/metastatic disease treated with anti PD–1 antibodies are planned. In future studies, we will also include the analysis of intracellular/nuclear PD–L1 expression, given the preliminary evidence of its interference with radiosensitivity [43] and outcome [44]. In addition, algorithms for the semi–automated quantification of CTCs with an activated EGFR pathway phenotype and γH2AX foci counting [9] will be optimized to further reduce intra- and interobserver variability of CTC analysis.

## 5. Conclusions

We successfully established a specific and sensitive assay for the detection and multiparametric phenotyping of CTCs using the Amnis^®^ ImageStream^®^X Mk II. We demonstrated the feasibility of our protocol for the analysis of intratumoral heterogeneity of PD–L1 expression, EGFR activation and the DNA damage repair in CTCs.

## Figures and Tables

**Table 1:** Patient characteristics.

		HNSCC	BC
**gender**			
**n**	female	4	8
	male	12	-
**age (years)**			
**median (range)**	female	70 (32–81)	48 (34–64)
	male	69 (58–79)	-
**stage of disease**			
**n (%)**	early stage	-	4 (50%)
	locally advanced	1 (6%)	-
	recurrent/metastatic	15 (94%)	4 (50%)
**tumor site**			
**n (%)**	oral cavity	8 (50%)	-
	oropharynx	3 (19%)	-
	hypopharynx	3 (19%)	-
	other/breast	2 (12%)	8 (100%)
**metastatic sites**			
**n (%)**	none	1 (0.06%)	4 (50%)
	regional	3 (25%)	3 (38%)
	distant	12 (75%)	1(12%)
**CTC^positive^ cases (≥ 3 CTCs)**			
**n (%)**		7 (44%)	6 (75%)
	**CTC numbers**		
	median	15	14
	range	6–30	9–27
**PD-L1^positive^ cases**			
**n (%)**		4 (57%)	4 (67%)
	**PD-L1^positive^ cells**		
	median (n)	6	2
	range (n)	3–30	1–6
**% of PD-L1^positive^ CTCs in entire CTC population**	median (%) range (%)	100% 56%–100%	15% 4%–25%

**Table 2:** **PD**–**L1 expression in tumor tissue and CTCs.** Results from cases with ≥ 3 CTCs and available matched tumor tissue are presented. Concordant cases are highlighted in green (TPS: tumor proportion score).

Pat.ID	Tumor Site	Date of tissue Biopsy (Month/Year)	Date of Liquid Biopsy (Month/Year)	PD–L1^positive^ Cells in Tumor Tissue (TPS %)	PD–L1^positive^ CTCs (%)	Time between Tumor and Liquid Biopsy (Months)
BC-003	breast	December/2017	December /2017	0	8	0.4
BC-006	breast	December /2017	January/2018	0	20	1
HNC-012	oral cavity	September/2017	September /2017	70	100	0.4
HNC-018	oral cavity	July/2016	January /2018	0	0	19
HNC-019	oral cavity	September /2017	January /2018	0	45	5
HNC-020	hypopharynx	January/2017	January /2018	5	100	12
HNC-026	hypopharynx	December./2016	Mar./2018	0	0	15

## Data Availability

The data that support the findings of this study are available from the corresponding author upon reasonable request.

## Supplementary Materials

The following supporting information can be downloaded at: https://www.mdpi.com/article/10.3390/cancers14112810/s1. Supplementary Table S1: Media composition used for cultivation of the mentioned cell lines. Supplementary Figure S1: Workflow of the CTC analysis. Supplementary Table S2: Laser settings. Supplementary Figure S2: Results from spiking experiments using the UD–SCC–4 cell line. Supplementary Figure S3: Quantitative analysis of γH2AX foci.
