# Supplementary material for: Multiparametric Phenotyping of Circulating Tumor Cells for Analysis of Therapeutic Targets, Oncogenic Signaling Pathways and DNA Repair Markers
Source: Cancers (Basel). 2022 Jun 6;14(11):2810. doi: 10.3390/cancers14112810 (PMC9179910; doi:10.3390/cancers14112810)
Supplement: Supplementary file 1 [file cancers-14-02810-s001.zip › cancers-1740537-supplementary.pdf]

Cell line	Culture media
**FaDu**<br>**UD-SCC-4**<br>**UM-SCC-22B**<br>(HNSCC)	**MEM Medium**<br>*(LifeTechnologies, cat. no.: 31095052)*<br>**12% FCS**<br>*(Life Technologies, cat. no.: 10270-106)*<br>**1x non-essential amnio acids**<br>*(LifeTechnologies, order: 11140035)*
**SCC-25**<br>(HSNCC)	**DMEM/F12 +GlutaMax**<br>*(ThermoFisher, cat. no.: 31331093)*<br>**10% FCS**<br>*(Life Technologies, order: 10270-106)*<br>**2mM L-Glutamine**<br>*(ThermoFisher, cat. no.: 25030149)*<br>**1% Penicillin/ Streptomycin**<br>*(Fisher BioReagent, cat. no.: BP2959-50)*<br>**15mM HEPES**<br>*(sigma, order: H4034-100g)*
**MDA-MB-231**<br>(TN breast cancer)	**Advanced DMEM/F12**<br>*(ThermoFisher, cat. no.: 12634010)*<br>**10% FCS**<br>*(Life Technologies, cat. no.: 10270-106)*<br>**2mM L-Glutamine**<br>*(ThermoFisher, cat. no.: 25030149)*
**SW620**<br>(colon cancer)	**RPMI Medium 1640**<br>*(LifeTechnologies cat. no.: 21875-034)*<br>**20% FCS**<br>*(Life Technologies, cat. no.: 10270-106)*<br>**2mM L-Glutamine**<br>*(ThermoFisher, cat. no.: 25030149)*<br>**1% Penicillin/ Streptomycin**<br>*(Fisher BioReagent, cat. no.: BP2959-50)*

Supplementary Table S1: **Media composition.**

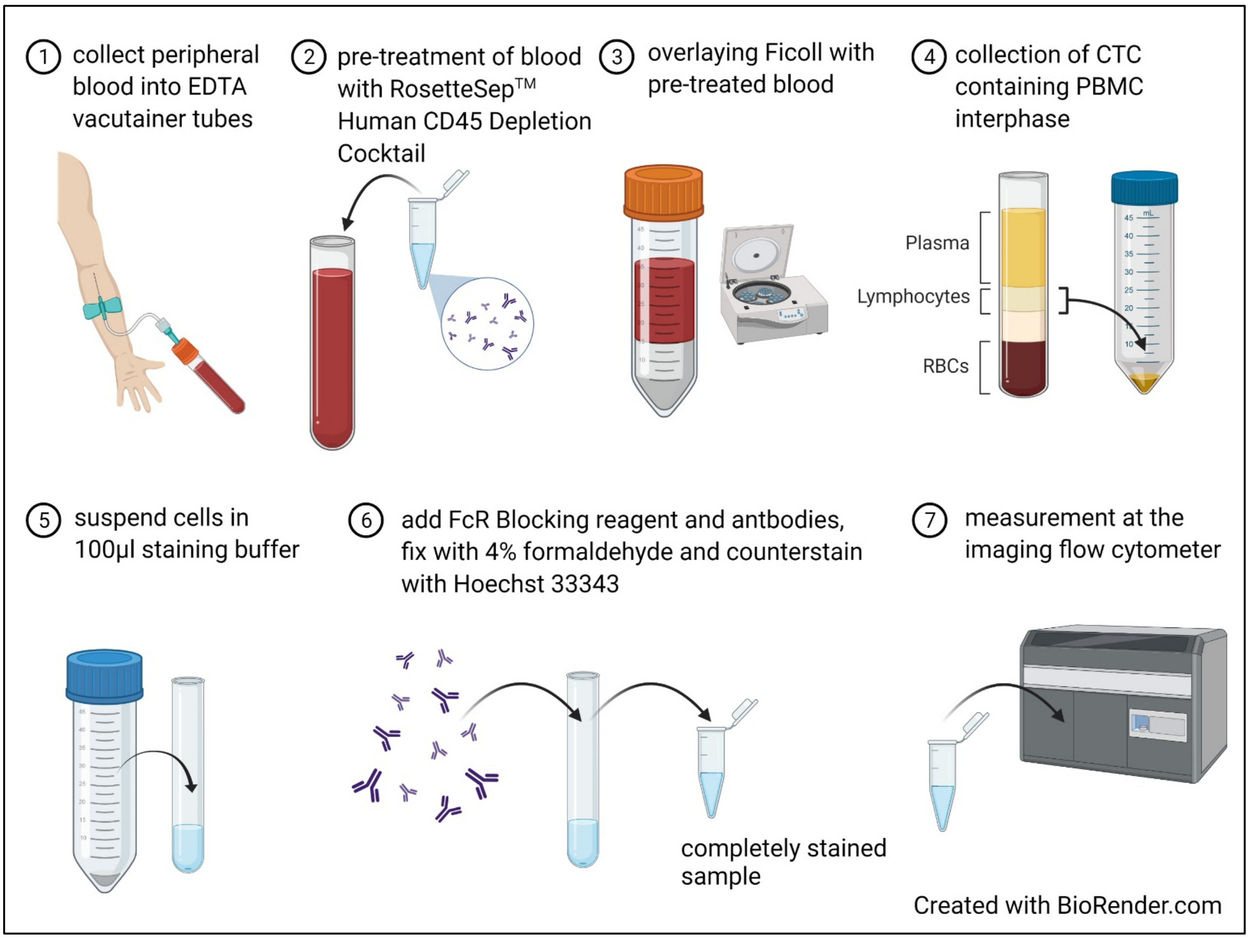

Supplementary Figure S1: Workflow of the CTC analysis. (1) Blood samples were processed within four hours after collection. (2) Enrichment of CTCs by adding RosetteSep™ Human CD45 Depletion Cocktail was done, (3) followed by Ficoll density centrifugation and (4) harvest of the PBMC interphase containing CTCs. (5-6) Next, cells were stained for extracellular markers, fixed with 4% formaldehyde and counterstained with Hoechst 33342 and (7) acquired at the Amnis® brand ImageStream®X MkII. ***Created with BioRender.com***

Laser (nm)	Target	Fluorophore	Channel	Laser power (mW)
405	Hoechst 33342	UV	7	30
488	EpCAM-EGFR	AF488	2	100
561	PD-L1	PE	3	200
561	phospho-EGFR	TexasRed	4	200
561	PD-L2	PE-Vio770	6	200
642	CD45 / γH2AX	AF647	11	150/130
642	CD45	APC/Fire	12	130
782	SCC		12	0.9
bright field			1 and 9	

Supplementary Table S2: Laser settings. For data acquisition the 40x magnification was used and the fluidics were set to low speed and high sensitivity, EDF-module was switched off and focus/ centering were set to automatic. CD45 signal was acquired in channel 12 (instead of 11) for samples stained for γH2AX.

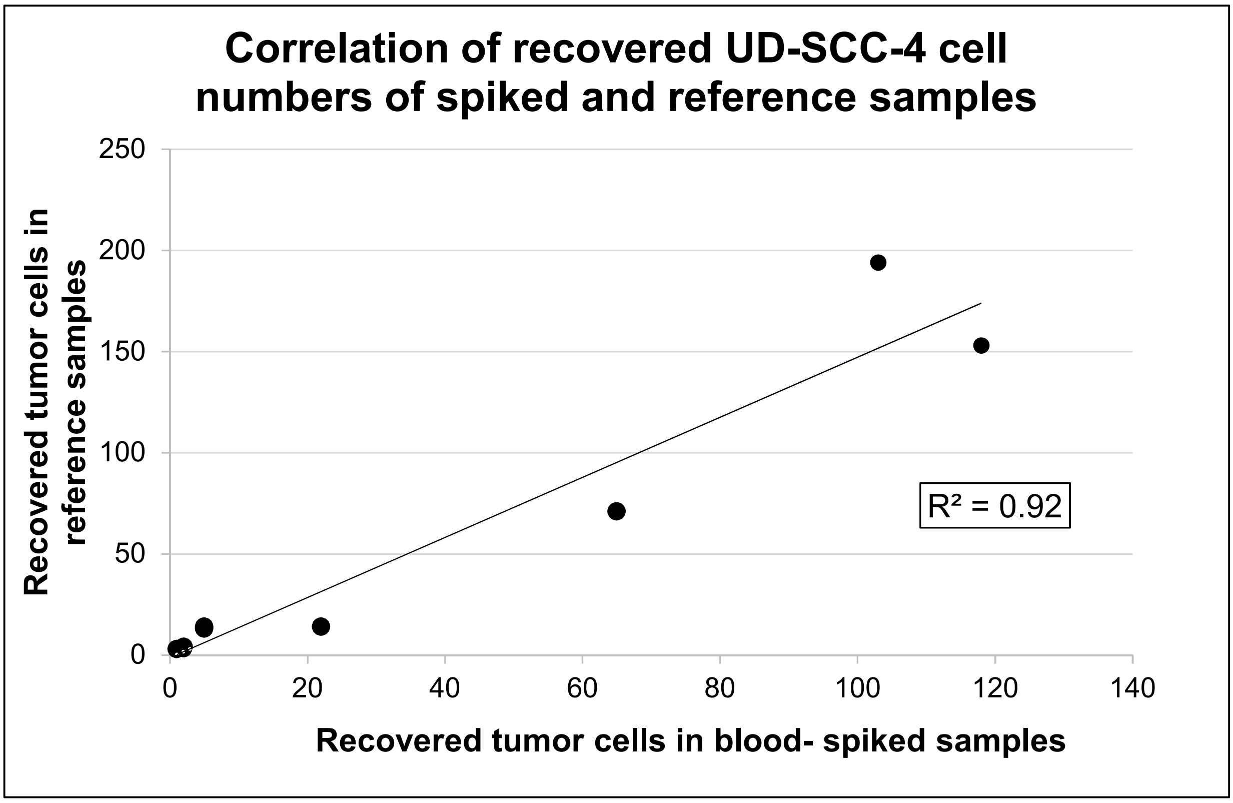

Supplementary Figure S2: Results from spiking experiments using the UD-SCC-4 cell line.

Analysis of samples (n=3 replicates per dilution) revealed a correlation of R^2= 0.92 of recovered cells in reference and spiked samples. The median recovery rate was 50%.

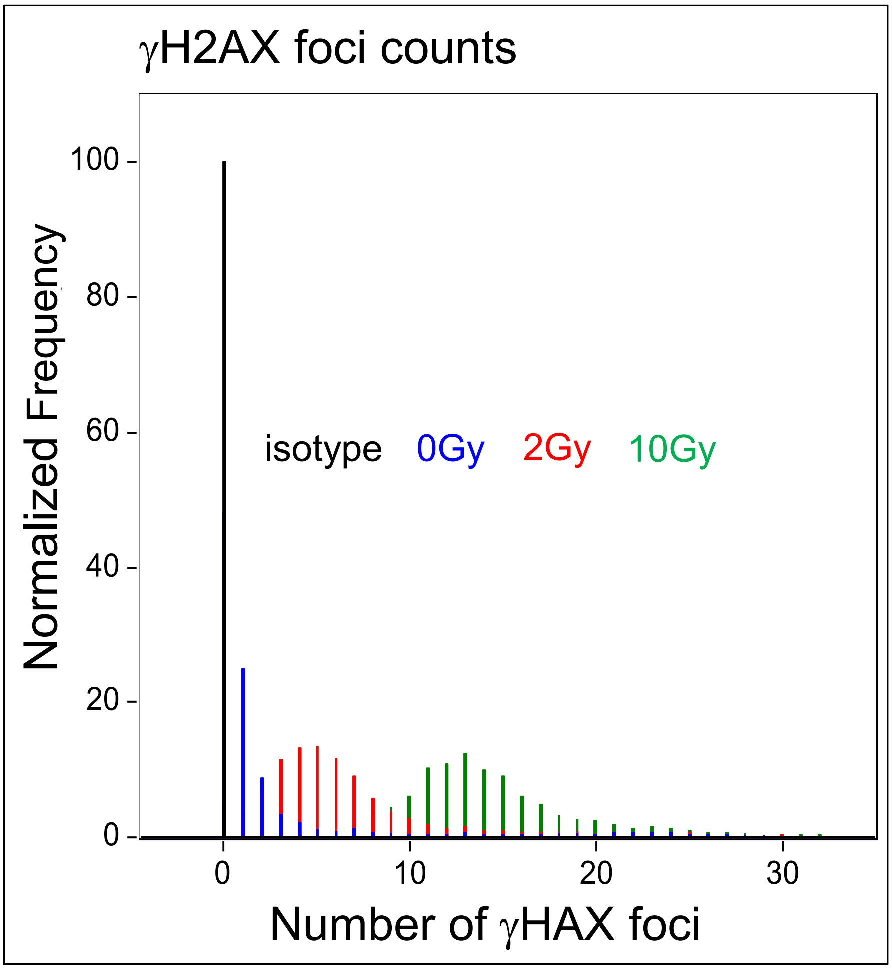

Supplementary Figure S3: Quantitative analysis of γH2AX foci. The distribution of the number of γH2AX foci detected in FaDu cells irradiated with the indicated dose is presented in the histogram.
